# Detection of leaf miner in sweet potato crops through image analysis using machine learning-based models

**DOI:** 10.3389/fpls.2026.1774493

**Published:** 2026-06-03

**Authors:** Brandon Huaman, Brayan Guzman, Juan Arcila

**Affiliations:** School of Systems Engineering, Universidad Señor de Sipán, Chiclayo, Peru

**Keywords:** deep learning, *Ipomoea batatas*, *Liriomyza huidobrensis*, pest detection, precision agriculture, YOLO

## Abstract

The leaf miner (Liriomyza huidobrensis) represents a critical threat to sweet potato production, causing irreversible damage that traditional visual inspection fails to mitigate efficiently due to its subjectivity and slowness. The objective of the present study was to automate the detection of this pest through image analysis using deep learning models. Methodologically, the dataset “camote_minador” was constructed and annotated, comprising 751 images collected from fields in Lambayeque, Peru, to which dynamic data augmentation techniques were applied to ensure training variability. The performance of the YOLOv8s and YOLOv11s architectures was comparatively evaluated under standardized hyperparameter configurations. The results demonstrated the technical superiority of the YOLOv11s model, which achieved a Precision of 73.20%, a Recall of 66.72%, and a mAP@50 of 71.63%, outperforming its predecessor and evidencing a greater ability to discriminate between pest galleries and background noise. Furthermore, the operational feasibility of a mobile prototype based on Tensor Flow Lite for mid-range devices was established. It is concluded that the implementation of optimized architectures such as YOLOv11s constitutes an effective, accessible, and scalable technological solution to strengthen phytosanitary monitoring in the agricultural sector.

## 1 Introduction

Agriculture, from ancient times to the present, is an activity dedicated to the production of various crops through their development and harvesting, to be subsequently distributed across different markets. However, these crops face significant risks due to challenges related to pest detection and control; such pests affect both the quality and quantity of agricultural products. Failure to detect these pests in a timely manner increases the likelihood of devastating impacts, as it significantly reduces crop yields and generates substantial economic losses for farmers (Al Hinaai et al., 2025).

In studies such as that reported in (Mujica and Kroschel, 2019), the intensive use of pesticides in crops across Latin America, particularly in Peru and Ecuador, is discussed, highlighting the negative impacts on the environment and providing an analysis of the effects on human health and farmers’ economic conditions. Integrated Pest Management (IPM) seeks to promote healthy and sustainable agricultural practices in the Cañete Valley through responsible pesticide management.

Likewise, in countries such as Brazil, research has analyzed the consequences of delayed manual inspection, the impact generated by pests on agriculture, and the technological needs that can be implemented for early pest detection (Dos Santos et al., 2018).

Pests are one of the key factors affecting agricultural production. The Food and Agriculture Organization of the United Nations estimates that pests cause between 20% and 40% of annual losses, reducing global production and representing a significant challenge for food cultivation (Balasubramaniam et al., 2024).

Another study (Khan et al., 2024), addresses the localization of diseases and pests in agricultural crops, indicating that the excessive use of pesticides can lead to pest resistance, making future applications less effective and forcing the use of more potent products and, in some cases, larger quantities. This generates environmental damage to soil, water, and other organisms, as well as health impacts on farmers and/or consumers if the pre-harvest interval is not respected, which is the time required for the product to dissipate before being suitable for consumption.

At the international level, one of the most important crops is sweet potato (Ipomoea batatas), which is positioned as one of the most prominent tropical root crops in terms of nutritional value. Worldwide, it is recognized as a significant contributor to food security, particularly in vulnerable regions. In the Latin American context, this tuber is part of the daily diet and culinary culture in several countries and is also of vital importance as a source of income within the agricultural sector in both Andean and coastal regions. However, sweet potato production also faces pests and diseases throughout its cultivation process. Even in improved cultivation systems, sweet potato and other agricultural products are affected by pests, requiring the application of improvements or the adoption of new strategies to achieve optimal crop yields (Kyalo et al., 2024).

During its cultivation process, sweet potato faces various threats that may affect the product at harvest time, among the main ones being the sweet potato weevil (Cylas formicarius), root-knot nematodes (Meloidogyne spp.), whitefly (Bemisia tabaci), sweet potato beetles (Euscepes postfaciatus), and the leaf miner (Liriomyza huidobrensis). The leaf miner, a pest that affects different crops such as coffee, can spread in a highly destructive manner across a wide range of crops, causing leaf desiccation, premature leaf drop, and damage to the product or fruit produced (Monteiro et al., 2021).

For this reason, leaf miner infestation causes severe damage to the foliar tissue of the plant, significantly reducing photosynthesis and affecting plant growth; consequently, both the quantity and quality of the harvested product are compromised.

Currently, the detection of leaf miner infestations is carried out through visual inspection; however, this process is slow and highly dependent on the experience of the personnel conducting the inspection. Additionally, these crops are not always cultivated in small areas; in many cases, they cover large land extensions, making plant-by-plant inspection ineffective. This situation limits early response capacity, favoring the spread of the insect within the crop before effective control measures can be implemented. Moreover, favorable environmental conditions further enable rapid pest propagation.

The lack of new solutions for rapid and accurate detection of the leaf miner leads to severe consequences, including reduced agricultural yield, increased pesticide use that deteriorates soil quality, adverse effects on consumer health, and, ultimately, significant economic losses. According to (Visscher et al., 2024), the direct economic impact of pests on crops is considerable, especially when their spread is rapid, resistant, and appropriate pesticides are not applied, resulting in substantial losses for farmers, which may represent 20% or more of total production.

To identify whether a plant is infested with the leaf miner, certain visual characteristics must be present on the leaf surface indicating damage to the plant. These include serpentine lines running through the interior of the foliar tissues, along with depigmentation along these lines, which represent the path left by the larva. Study (Math and Dharwadkar, 2023) classifies these infestations into stages based on the level of damage caused to the leaf, such as low, medium, and high; this characteristic is key to identifying the progression of infestation relative to the plant.

Timely detection of the aforementioned pest not only compromises agricultural sustainability but also reflects the technological gap within the sector, highlighting needs that should be prioritized to incorporate state-of-the-art tools such as Machine Learning (ML) to strengthen pest monitoring and control systems.

In this regard, studies in computer science have driven significant advances in early pest identification. For example, research such as that presented in (Vilela et al., 2023) has demonstrated the effectiveness of image analysis–based systems in facilitating pest identification, enabling the detection of features related to their presence with greater accuracy than conventional methods.

Nevertheless, a deficit persists: the application of new technologies entails unforeseen expenses for farmers, as well as a lack of knowledge regarding their use. Paraphrasing a (Huarancca et al., 2024), the slow adoption of technology in the agricultural sector depends on factors such as limited financial resources, insufficient farmer training, and resistance to change regarding new tools for agricultural development. Despite these factors, limited access to advanced technologies and precise data for agricultural decision-making results in continued reliance on traditional methods.

Within the previously described context, delayed detection of leaf miner infestations in sweet potato crops not only leads to losses but also degrades the intrinsic quality of the sweet potato itself. Although traditional methods are effective up to a certain point, they present major limitations in current inspection practices, making evident the need for technological innovations that enable precise detection. As highlighted in (Mahlein, 2016), the use of anomaly recognition technologies in images for pest detection is fundamental to reducing agricultural losses by improving accuracy and efficiency in early pest containment.

Given this scenario, the need to implement new strategies that integrate modern technologies such as ML and image analysis becomes apparent. Their combination offers a real opportunity to strengthen monitoring systems, reduce response times, decrease pesticide use that negatively affects human health, and preserve environmental quality. The use of Artificial Intelligence in agriculture allows for significant improvements in disease tracking within agricultural environments and supports informed decision-making for pest mitigation (Kamilaris and Prenafeta-Boldú, 2018).

**Table 1** presents various studies focused on the automatic recognition of agricultural pests and diseases, along with the methods used, data sources, and results obtained.

**Table 1:** Studies applying techniques for the identification and classification of agricultural.

Technique	Context	Dataset	Results
CNN (ResNet-18) with transfer learning from ImageNet (Kini et al., 2024)	Detection of pathologies in pepper leaves with field images validated by experts	Field images, early disease stages.	Precision 99.67%; high accuracy in disease detection
CNN and M5C and M10C models; robotics and virtual databases (Liu and Chahl, 2021)	Detection of invertebrate pests; incorporates robotics and virtual databases	Real data with virtual databases	CNN 97.8%; M5C 97.89%; M10C 65.44% — reliable models depending on data quantity
Image enhancement (contrast) and ML: Decision Tree, CNN, ResNet (Chand et al., 2023a)	Whitefly detection with size and lighting variations	Crop images in diverse conditions	Tree 81%; CNN 96%; ResNet 97.5% image enhancement increases precision
MA, CNN, LSTM and variants (integrated with IoT) (Padmavathi et al., 2024)	Predictive framework for classification and prediction of diseases and pests (temporal data)	Crop data and IoT sensors (time series)	Classification precision 94.17%; improvement over traditional methods
CNN with Dropout and ELU activation (Jabir and Falih, 2021)	Weed detection; regularization to reduce overfitting	Weed/crop images	Reduced overfitting and better precision when using dropout + ELU
Bayesian Network, CNN, ResNet, Multi-Instance Learning–CNN (Chand et al., 2023b)	Comparative classification of whitefly infestations.	Whitefly images	Precision: 95.53% (Bayesian); 98.13% (MIL–CNN) more precise
MMTL-IPCAC (CLAHE + NASNet + MGWO + XGBoost) (Yonbawi et al., 2023)	Insect classification with image enhancement and hyperparameter tuning	Images processed with CLAHE; extraction with NASNet	Precisión 98,73%; clasificación confiable.
Deep detection models for counting and localization (improvement over YOLOv4 and Faster R-CNN) (Ciampi et al., 2023)	Insect counting and localization in traps for integrated pest management	Annotated trap images	Average error ≈ 9% in population estimation; better spatial accuracy
Modified YOLOv5s (expanded CSP, improved SK) (Aladhadh et al., 2022)	Detection of five harmful pests with a practical approach for agricultural production	Custom set with five pest classes	Performance validated for practical application
YOLO variants (v5 n/s/l/x, v3, Lite, YOLOR) on smartphone with IP camera (Ahmad et al., 2022)	Smartphone system (IP camera) to identify pests and reduce pesticides	IP-23: 7,046 images in varied conditions	YOLOv5x: mAP@0.5 = 93.3%; rapid and precise detection.

The present research is justified by the need to incorporate modern technologies into the agricultural domain, particularly for the early detection of pests such as the leaf miner affecting sweet potato crops. The use of machine learning techniques combined with image analysis offers an efficient alternative to traditional manual inspection methods, which are slow, subjective, and ineffective over large cultivation areas. This proposal aims to optimize the use of agricultural resources, reduce the negative impact of excessive pesticide use on human health and the environment, and increase the sustainability and productivity of crops.

The general objective of this research was to detect the leaf miner in sweet potato crops through the analysis of plant images using Machine Learning–based models. To achieve this purpose, the following specific objectives were defined: to design a database with the images required for model preparation; to prepare and train the Machine Learning model using the dataset images; to evaluate the models using standard metrics such as precision, sensitivity, and specificity; and to develop a functional prototype that integrates the trained model for automated detection of the leaf miner in sweet potato leaves. In line with these premises, the hypothesis guiding this study posits that by using Machine Learning, based models, it is possible to effectively detect the presence of the leaf miner through image analysis of sweet potato plants.

## 2 Data and materials

### 2.1 Dataset

Image acquisition was conducted *in situ* within commercial crop fields located in the Department of Lambayeque, Peru, selecting two strategic geographic zones to capture the crop’s phenological variability. The first sampling site was located in the Chosica del Norte sector (Latitude: -6.8361°, Longitude: -79.8345°), on a crop at 3 months of physiological maturity; while the second site corresponded to the Callanca sector (Latitude: -6.8367°, Longitude: -79.8168°), in a plantation at 2 months of growth. The inclusion of crops at different stages of vegetative development and data capture under open-field conditions allowed for the incorporation of natural variability factors such as changes in foliar density, uncontrolled solar lighting, and the presence of dust. This distinguishes this study from those based on laboratory environments, ensuring that the model learns to discriminate leaf miner symptoms under real agricultural operating conditions.

**Figure 1:**
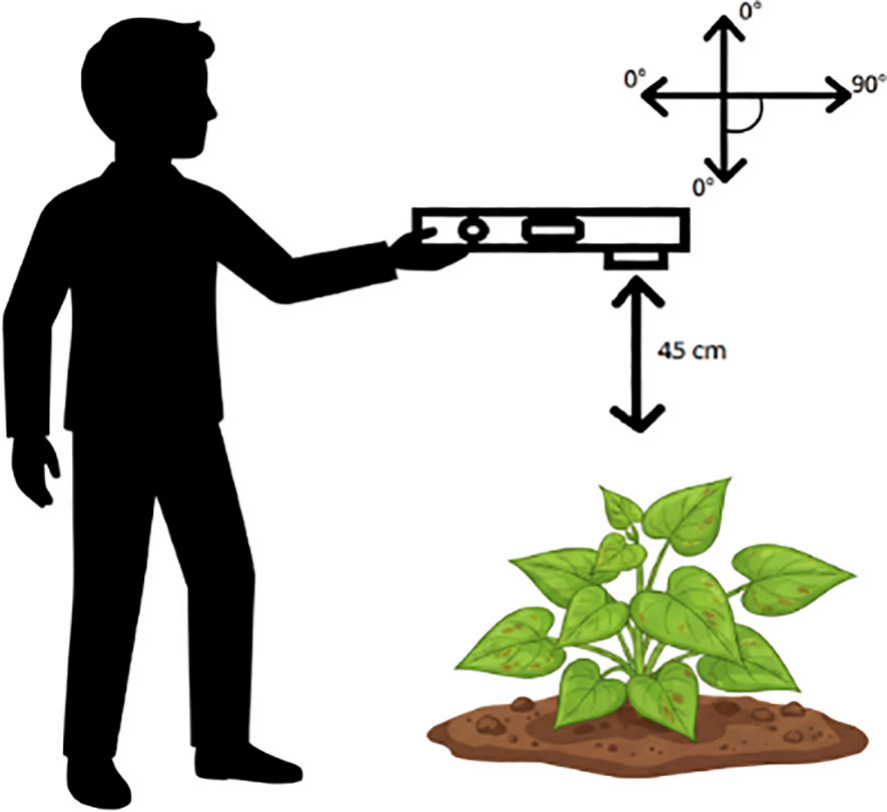
Image capture model.

To ensure the consistency and replicability of the study, a protocol was established with the following standard for image acquisition. The technical specifications of the devices and the capture settings are detailed in **Table 2**. For a better understanding of the acquisition method, **Figure 1** illustrates the image capture procedure, showing the relative position of the device with respect to the sweet potato plant and the perpendicular angle employed, which are key aspects for obtaining images with uniform quality and avoiding distortions.

**Table 2:** Specifications of the standardized protocol for sweet potato image capture.

Criterion	Description
Capture Devices	Redmi Note 11 (50 MP camera)
	Redmi Note 13 Pro (50 MP camera)
Resolution	4080 x 3072
Height (Min. shooting distance)	45 cm relative to the foliar surface of the sweet potato plant
Capture Angle	90° to the foliar surface
Digital Zoom	Not employed
Selection Criterion	Economic accessibility for the farmer is prioritized
Image Capture Dates	June 24, 2025 (3-month crop)
	July 30, 2025 (2-month crop)

**Figure 2:**
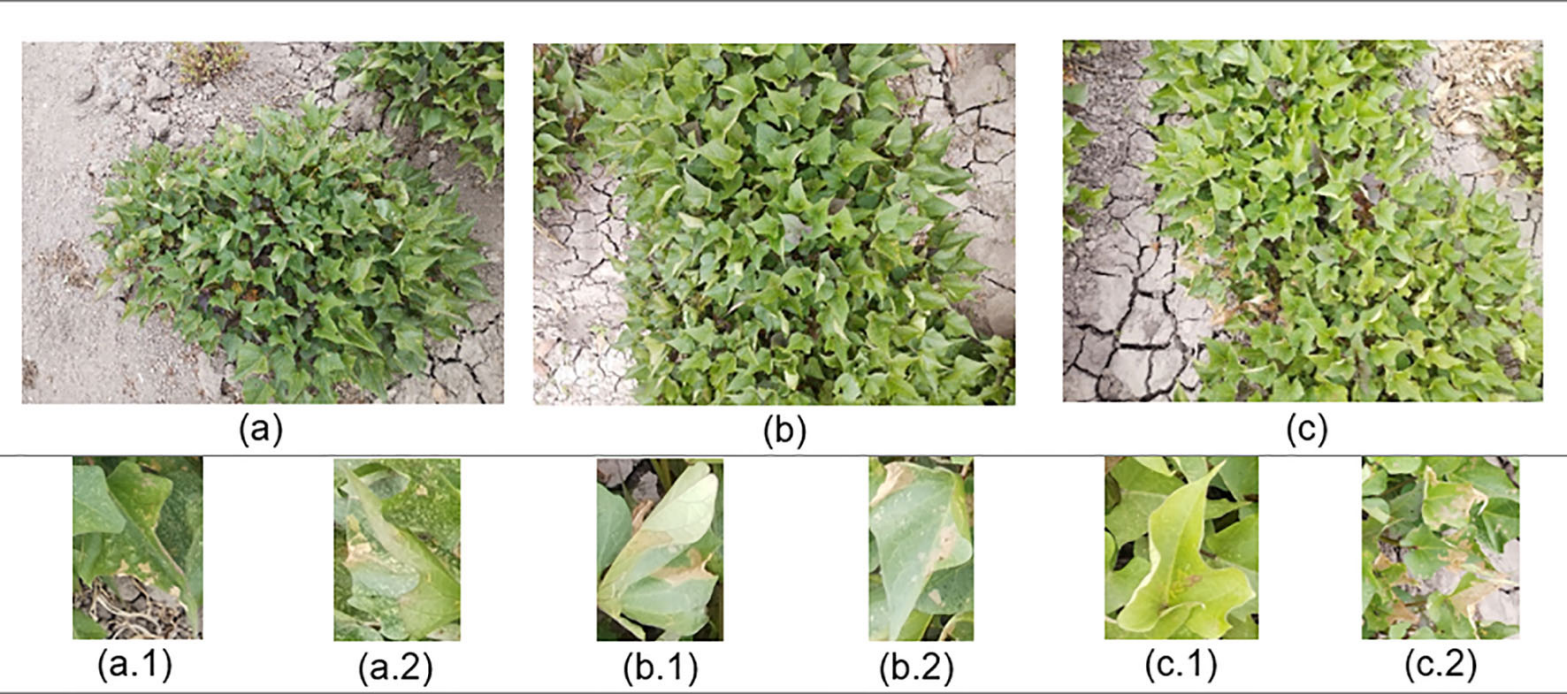
Representative samples of the dataset showing leaf miner damage. **(a–c)** General views of the sweet potato crops from different perspective angles and distances, and **(a.1–c.2)** detailed foliar views displaying representative damage characteristics on the leaves.

A total of 350 images with dimensions of 8160 × 6144 px were captured using Redmi Note 11 and Note 13 Pro devices (50 MP cameras). However, these images were excessively large for the model to accurately localize the leaf miner; therefore, the images were cropped and resized to a resolution of 1280 × 1280 px, resulting in a total of 751 cropped images. All images were stored in JPG format. **Figure 2** schematically illustrates this organization process, from image acquisition to the structuring of the established dataset. Images (a), (b), and (c) illustrate sweet potato plants infested by the leaf miner, while insets (a.1) to (c.2) detail the specific damage to the foliar tissue.

For the development of this research, the following tools were employed, selected for their accessibility, computational efficiency, and compatibility with YOLO, thereby ensuring the reproducibility of the study, as shown in **Table 3**, which lists the selected tools.

**Table 3:** Tools used.

Category	Tools	Component / Tool	License	Description
Hardware	Capture devices	Redmi Note 11 (50 MP camera)	Proprietary	Devices selected for their economic accessibility and high-resolution capture capability.
		Redmi Note 13 Pro (50 MP camera)		
	Training platform	Google Colab Pro	Commercial Subscription	With NVIDIA A100 GPU with 40 GB VRAM for deep learning model training
Software and platform	Image Annotation	Labelmg 1.21.0	MIT	For image labeling with bounding boxes
	Programming language	Python 3.10	Python license	Main programming language for implementation and analysis
	Library	PyTorch 2.9.0	BSD license	Deep learning base library for the YOLO architecture.
	Python Libraries	OpenCV 4.13.0		Image processing and visualization
		NumPy 2.0.2		Mathematical operations and array manipulation
		Pandas 2.2.2		Tabular data analysis and metrics

### 2.2 Model selection

The study population is composed of algorithms applied to disease detection in crops through image analysis. Among them, Convolutional Neural Networks (CNNs) stand out, which through the analysis of smartphone-captured images, have achieved high accuracy in the detection and classification of various foliar pests, enabling robust automated identification (Fallah and Parmehr, 2023). Similarly, YOLO (v8) demonstrated high real-time effectiveness in identifying various insects, achieving an mAP@50 of 0.967 (Vilar-Andreu et al., 2024). Random Forest attained 98% accuracy by analyzing features such as texture and color in soybean leaves (Mahobiya et al., 2023), while ResNet (ResNet50) optimized visual classification through self-attention mechanisms, achieving 99% accuracy (Hassan and Maji, 2024). Finally, models such as LSTM are considered, with a precision of 98.50% in temporal sequence analysis (Al-Shahari et al., 2024), and XGBoost, which achieved 99.43% accuracy in pest detection (Arasi et al., 2024).

The sample consists of a representative subset of machine learning algorithms that have demonstrated high levels of accuracy in pest detection tasks in crops using digital images, as detailed in **Table 4**.

**Table 4:** Sample of high-performance algorithms for pest detection.

Algorithm	Precision (%)	Data	Studies
YOLO	64.7 ± 97.6	Handheld camera images (RGB type)	(Ciampi et al., 2023) (Ahmad et al., 2022) (Aladhadh et al., 2022)

## 3 Method

**Figure 3:**
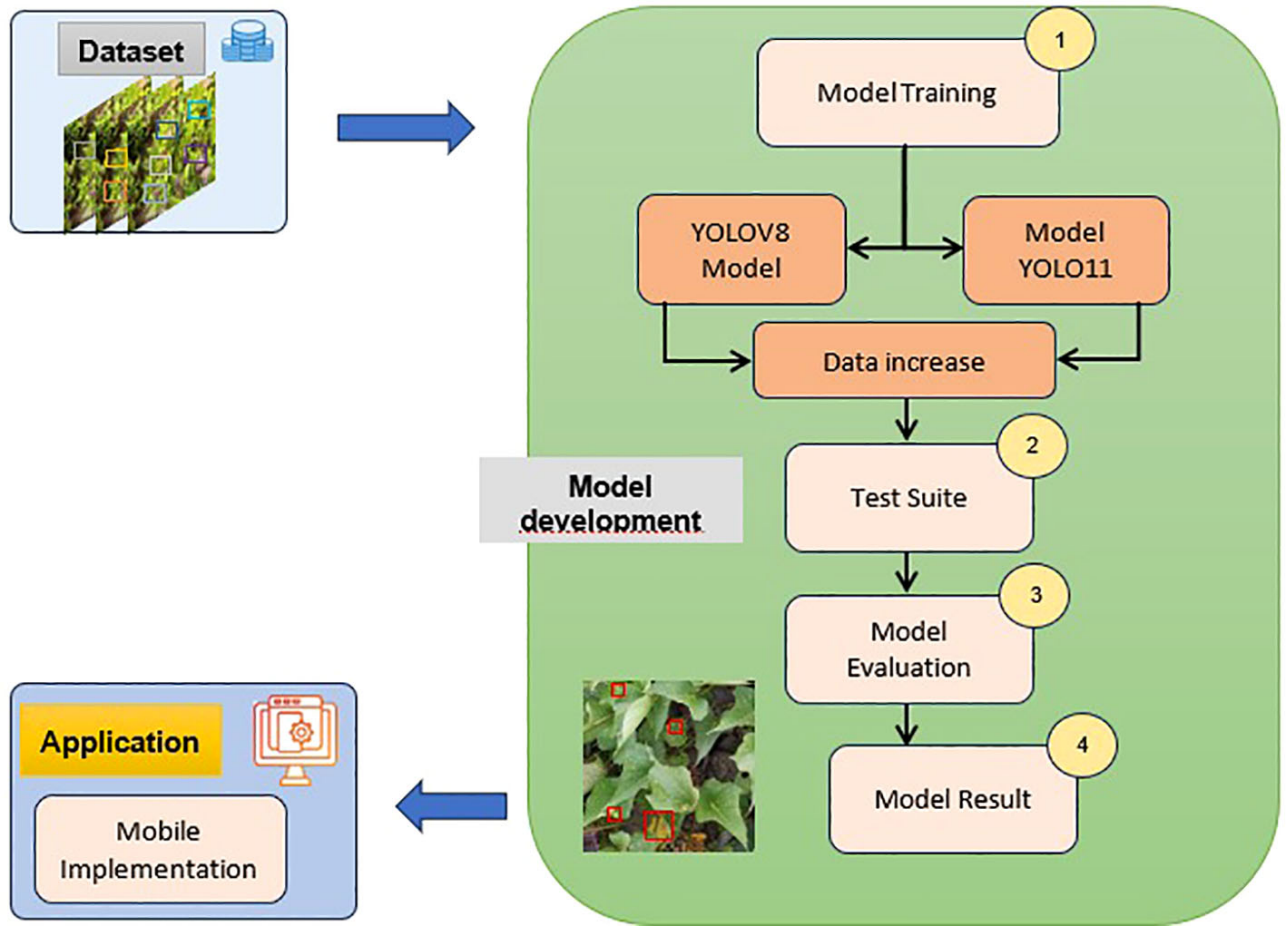
Sequence of methodological development.

**Figure 3** shows the methodological sequence developed in this research for the detection of the leaf miner in sweet potato crops, using machine learning models based on the YOLO architecture.

### 3.1 Dataset preprocessing

Proper dataset preparation constitutes a determining factor in the performance of deep learning–based object detection models. This stage comprises the annotation of the captured images, the stratified division of the dataset into training, validation, and test subsets, and the application of preprocessing techniques to ensure compatibility with the YOLO architecture.

#### 3.1.1 Bounding box annotation

The manual annotation of the 751 images was carried out using the local web-based platform “Label Studio”, employing rectangular bounding boxes to mark foliar areas that exhibited visual indicators of infestation caused by the leaf miner. These regions corresponded to serpentine galleries, necrotic sectors, and areas with characteristic discoloration produced by pest damage.

Regarding the YOLO annotation format, each bounding box was represented using normalized coordinates with respect to the total image dimensions. The coordinates of the center point of each box were determined using Equations (1, 2).

(1) $$x_{center}=\frac{x+\frac{w}{2}}{Width_{image}}$$

(2) $$y_{center}=\frac{y+\frac{h}{2}}{Height_{image}}$$

**X:** Indicates the horizontal coordinate (x) of the upper-left corner of the bounding box.

**Y:** Indicates the vertical coordinate (y) of the upper-left corner of the bounding box.

**w:** Width of the bounding box.

**h:** Height of the bounding box. $Width_{image}$: Total image width. $Height_{image}$: Total image height.

The normalized dimensions of the bounding box were obtained by applying Equations (3, 4).

(3) $$w_{norm}=\frac{w}{Width_{image}}$$

(4) $$h_{norm}=\frac{h}{Height_{image}}$$

The resulting label file for each image consisted of a plain text file (.txt) structured according to the standard YOLO format: <class_id> <x_center> <y_center> <w_norm> <h_norm>. To this end, a binary encoding was defined wherein class 0 (‘Leaf miner’) delimits the foliar area exhibiting the characteristic galleries of the pest. This distinction enables the model to effectively discriminate between the distinct foliar tissue conditions during training.

#### 3.1.2 Dataset splitting

The dataset distribution was carried out through stratified partitioning to ensure proportional representativeness of the different classes in each subset. A standard split widely used in the computer vision literature was adopted: 70% for training, 15% for validation, and 15% for testing, out of the total of 751 images.

#### 3.1.3 Image preprocessing

Prior to training, the images underwent a standardized preprocessing procedure to ensure dimensional compatibility with the YOLO architecture and to optimize model convergence. The applied transformations included uniform resizing and pixel intensity normalization.

**Figure 4:**
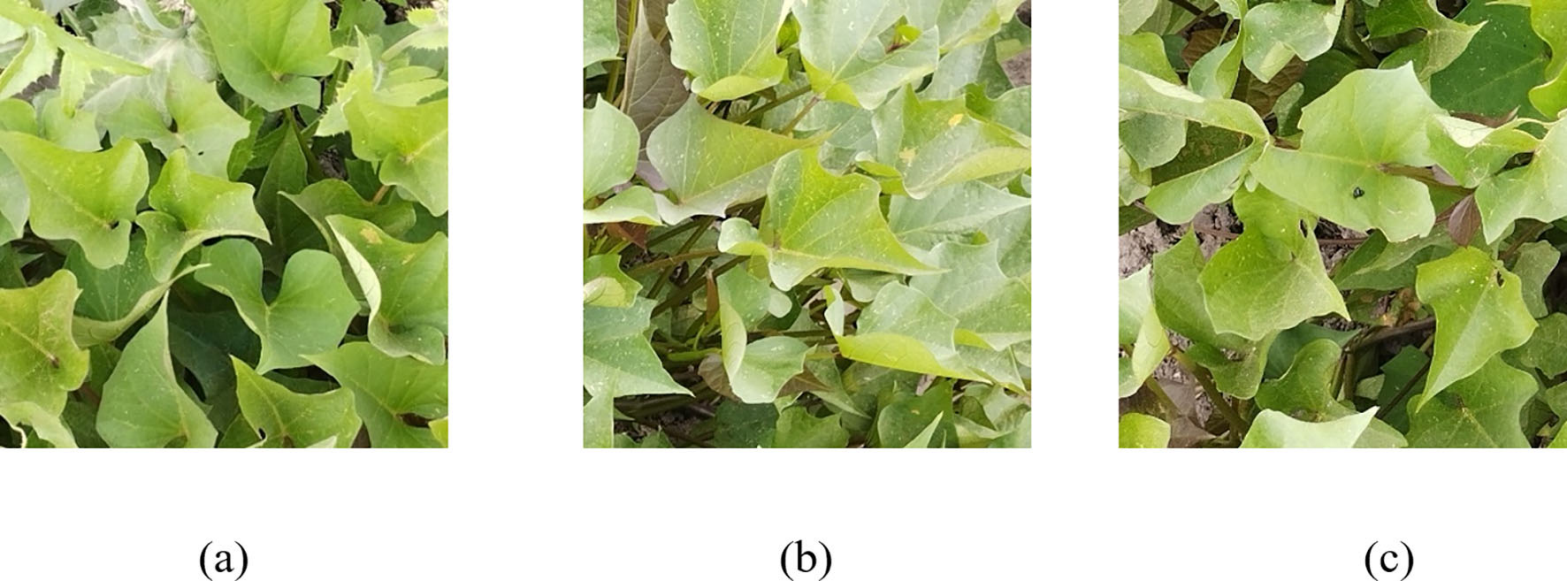
Example images from the dataset utilized for model training and evaluation. **(a–c)** Representative samples of sweet potato foliar images captured under field conditions, showcasing different lighting variations and leaf positions.

All images were standardized to a resolution of 1280 × 1280 pixels, a configuration specifically selected for training in order to maximize the detection of small details. **Figure 4** presents representative examples of the dataset processed under these dimensions, allowing the morphology of foliar damage to be observed while avoiding distortions that could affect model learning.

Subsequently, pixel intensity values, originally in the range [0, 255] for 8-bit RGB images, were normalized to the interval [0, 1] through simple division by 255. This normalization facilitates numerical stability during forward propagation and gradient computation, accelerating model convergence and reducing the risk of issues associated with excessively large activation values.

It should be noted that, unlike traditional approaches that apply data augmentation through the synthetic generation of new images prior to training, in this study data augmentation techniques were applied dynamically during the training process. This strategy, integrated into the YOLO pipeline, allows each epoch to present random variations of the original images, maximizing the diversity of the training set without increasing storage requirements.

### 3.2 YOLO model training

#### 3.2.1 YOLOv8 model

##### 3.2.1.1 Architecture selection

For the development of this detection system, the YOLOv8s (Small) architecture from the YOLOv8 family, developed by Ultralytics, was selected. This variant represents an optimal balance between detection accuracy and computational efficiency, which are critical aspects for subsequent deployment on mobile devices with limited resources. YOLOv8s comprises 11.2 million trainable parameters and requires 28.6 GFLOP to process a 640 × 640 pixel image, enabling theoretical speeds of approximately 100 frames per second (FPS) on mid-range hardware.

However, the experimental configuration of this study employs a resolution of 1280 × 1280 pixels, which increases the theoretical computational load to approximately 114.4 GFLOP. Although this modification does not alter the 11.2 million structural parameters of the model, it entails a necessary reduction in frame rate (FPS) in exchange for the visual acuity required to identify leaf miner galleries.

The YOLOv8s architecture belongs to the category of one-stage object detectors, performing class classification and bounding box coordinate regression simultaneously in a single forward pass through the neural network. This characteristic is particularly advantageous for the detection of small objects such as leaf miner galleries, which typically occupy less than 5% of the total area of the captured image. The network backbone is based on a modified version of CSPDarknet53, optimized with residual connections (skip connections) that facilitate gradient flow during training and enhance multi-scale feature extraction, enabling effective detection of objects of varying sizes and positions within the image.

##### 3.2.1.2 Data augmentation

In order to increase the diversity of the training set and improve the model’s generalization capability under varying capture conditions, a robust set of data augmentation techniques was implemented and applied dynamically during training. Unlike offline augmentation, where modified versions of images are generated and stored prior to training, online augmentation applies random transformations in real time during each training epoch, allowing the model to observe different synthetic variations at each iteration without increasing storage requirements. The data augmentation strategy was configured to apply dynamic transformations in each epoch. Image mixing techniques such as Mosaic (probability of 1.0) were prioritized to expose the model to multiple contexts simultaneously, and Mixup (alpha 0.15) to smooth decision boundaries through implicit regularization. Additionally, Copy-Paste was employed with a probability of 30% to increase instance density in complex scenarios. To simulate the geometric variability of the field, random rotations within a range of ±25° and horizontal and vertical flips with a probability of 50% were applied. Finally, differences in photographic exposure were compensated for through variations in the HSV color space, applying a gain of 0.4 to the value (brightness) channel.

Among the applied geometric transformations, rotation stands out for its mathematical relevance, as it involves the direct modification of the spatial coordinates of each pixel. Random rotations were determined according to (Equation 5)

(5) $$I_{rot}(x^{\prime },y^{\prime })=I(x\cos(\theta )-y\sin(\theta ),x\sin(\theta )+y\cos(\theta ))$$

I: represents the original image

$I_{rot}$: denotes the rotated image

(x,y): represent the original pixel coordinates

$\left(x^{\prime },y^{\prime }\right)$: represent the transformed coordinates

θ: rotation angle

This transformation, together with the other applied techniques, preserves the morphological structure of foliar damage (serpentine galleries, discoloration areas, necrotic zones) while incorporating variations in scale, orientation, illumination, and focus level that simulate real capture conditions in uncontrolled field environments.

##### 3.2.1.3 Training configuration

The training process was configured using the Ultralytics YOLO framework implemented on PyTorch 2.x, with an optimization strategy based on Adaptive Moment Estimation with Weight Decay (AdamW). This optimizer combines the advantages of adaptive momentum with explicit regularization through weight decay, proving particularly effective in preventing overfitting in moderately sized datasets such as the one used in this research. The initial learning rate was set to 0.001, with a cosine decay schedule that progressively reduces the value to 0.01 over the planned 150 epochs, allowing broad initial exploration of the parameter space followed by fine-tuning in the final stages.

The loss function weights were adjusted to prioritize precise localization of small objects, a critical characteristic given the nature of leaf miner galleries. A weight of 7.5 was assigned to the bounding box regression loss (box loss), 0.5 to the classification loss (cls loss), and 1.5 to the distribution focal loss (dfl loss), which refines coordinate prediction through probability distributions. The total loss function is expressed as shown in (Equation 6).

(6) $$L_{total}=\lambda_{box}.L_{box}+\lambda_{cls}.L_{cls}+\lambda_{dfl}.L_{dfl}$$

Additionally, an early stopping mechanism with a patience of 20 epochs was implemented, monitoring the mAP@0.5 on the validation set; training was automatically halted if no improvement was observed for 20 consecutive epochs. This strategy prevents overfitting and reduces unnecessary computational time. **Table 5** summarizes the complete hyperparameter configuration of the model.

**Table 5:** Main hyperparameter settings for training YOLO v8s and v11s.

Hyperparameter	YOLOv8s	YOLOv11s	Description
Epochs	150	150	Maximum number of complete iterations over the training dataset.
Batch Size	24	16	Number of images processed simultaneously in each iteration.
Resolution	1280×1280 px	1280×1280 px	Input image dimensions.
Optimizer	AdamW	AdamW	Adaptive Moment Estimation con Weight Decay.
Box Loss Weight	7.5	7.5	Weight of the bounding box regression loss function.
Workers	8	8	CPU threads for parallel data loading; accelerates the preprocessing pipeline.

#### 3.2.2 YOLOv11 model

##### 3.2.2.1 Architecture selection

With the aim of exploring the most recent architectural improvements within the YOLO family, the YOLOv11s (YOLO11 Small) model was additionally selected. This version, released by Ultralytics in 2024 as a direct evolution of YOLOv8, incorporates specific innovations oriented toward small object detection, a critical characteristic for the leaf miner use case, as its foliar galleries frequently occupy less than 5% of the captured image area. YOLOv11s comprises 9.4 million trainable parameters and requires 21.5 GFLOP to process a 640 × 640 pixel image, representing a 16% reduction in parameters and a 25% reduction in computational load compared to YOLOv8s, while maintaining or surpassing its detection capability.

The base architecture of YOLOv11s is optimized for 21.5 GFLOP under a standard input resolution. However, in this research, an input resolution of 1280 × 1280 pixels was configured to maximize the detection of leaf miner galleries. This strategic decision preserves the 9.4 million model parameters unchanged but increases the theoretical computational load to approximately 86 GFLOP, prioritizing spatial accuracy over the maximum theoretical speed of 200 FPS.

The main architectural innovations of YOLOv11s are concentrated in three fundamental aspects. In the backbone, the C2f blocks of YOLOv8s are replaced by C3k2 (Cross-Stage Partial with k = 2) blocks, which incorporate hybrid convolutional kernels of sizes 3 × 3 and 5 × 5, enabling the capture of visual patterns at multiple spatial scales within a single block. This characteristic is particularly advantageous for detecting the serpentine galleries of the leaf miner, which may exhibit variable widths depending on the larval stage of the insect. In the neck, the traditional PAN architecture is replaced by the Cross-Stage Partial with Spatial Attention (C2PSA) module, which integrates spatial attention mechanisms to selectively focus on regions of the image with a higher probability of containing objects of interest, thereby reducing the influence of irrelevant background areas such as exposed soil or crop support structures. Finally, the detection head incorporates the Spatial Pyramid Pooling with Efficient Layer Aggregation Network (SPPELAN) module, an optimized version of conventional SPP that enhances multi-scale feature extraction through pyramidal pooling with efficient connections.

##### 3.2.2.2 Data augmentation

In order to ensure a controlled comparison between both architectures and to isolate the effect of the architectural improvements of YOLOv11s from potential differences in data preprocessing, identical data augmentation techniques to those described for YOLOv8s were applied. This included the use of geometric transformations (Mosaic with a probability of 1.0, Copy-Paste with a probability of 0.3, rotations of ±25°, and horizontal and vertical flips with a probability of 0.5 each) and photometric transformations (HSV-H variations of 1.5%, HSV-S of 70%, and HSV-V of 40%), all applied dynamically during each training epoch.

This methodological decision ensures that any differences observed in performance metrics between YOLOv8s and YOLOv11s can be attributed exclusively to the architectural characteristics of each model, rather than to variations in dataset processing.

##### 3.2.2.3 Training configuration

The training process of YOLOv11s followed a hyperparameter configuration consistent with that employed for YOLOv8s, with a critical modification aimed at improving small object detection: the input resolution was increased from 640 × 640 pixels to 1280 × 1280 pixels. This decision is based on the fact that leaf miner galleries, after standard resizing to 640 pixels, can be reduced to areas smaller than 32 × 32 pixels, a size at which even modern architectures encounter detection difficulties. By doubling the input resolution, a greater amount of fine-grained visual information corresponding to the characteristic patterns of foliar damage is preserved, allowing the model’s convolutional layers to extract richer and more discriminative features. The main hyperparameters are shown in **Table 5**.

### 3.3 Model evaluation

The performance evaluation of both YOLOv8s and YOLOv11 models was conducted on the test set comprising 114 images that were not seen during training or validation. The following standard object detection metrics were computed.

Precision: quantifies the proportion of correct detections relative to the total number of detections performed by the model. A detection is considered correct when the Intersection over Union (IoU) between the predicted bounding box and the ground truth is greater than or equal to 0.5. It is calculated using (Equation 7).

(7) $$Precision=\frac{TP}{TP+FP}$$

Where:

TP: True Positives

FP: False Positives

This metric allows evaluating how many of the predictions labeled as infested were actually correct. In the context of leaf miner detection in sweet potato leaves, this metric is essential to prevent the system from incorrectly classifying healthy plants as infested.

Recall: Measures the proportion of actual objects that were correctly detected by the model. (Equation 8) shows how it is defined.

(8) $$Recall=\frac{TP}{\left(TP+FN\right)}$$

Where:

TP: True Positives

FN: False Negatives

It represents the number of real objects not detected by the model. A high recall indicates that the model detects most of the objects present in the images, minimizing cases of undetected infestation.

F1-Score: Provides a balanced metric that combines Precision and Recall through their harmonic mean, penalizing extreme imbalances between them. It is calculated using (Equation 9).

(9) $$F1-Score=2\frac{\left(Recall*Precision\right)}{\left(Recall+Precision\right)}$$

The F1-Score reaches its maximum value (1.0) when both Precision and Recall are perfect, making it particularly useful for evaluating the overall performance of the model.

Mean Average Precision (mAP@0.5): This is the primary metric used in object detection with YOLO. It is calculated as the area under the Precision–Recall curve with an IoU threshold of 0.5. For this binary classification study with a single class, mAP@0.5 coincides with the Average Precision of that class.

mAP@0.5:0.95: This is a more stringent metric that computes the average mAP over 10 IoU thresholds ranging from 0.5 to 0.95 in increments of 0.05. This metric penalizes detections with imprecise spatial localization and is particularly relevant when high accuracy in bounding box coordinates is required. .

## 4 Results

The results of the comparative analysis between the YOLOv8 and YOLOv11 models, both in their “s” (small) architectures, are presented below. The evaluation is based on standard metrics (mAP@50, precision, and recall) and their corresponding learning curves.

### 4.1 Overall performance analysis

To ensure a fair comparison, a training protocol of 150 epochs was established for both architectures using the same hyperparameters. However, learning convergence was observed before reaching this limit, and the early stopping (ES) mechanism terminated both training processes at epoch 79. This indicates that both models stabilized their learning processes and reached their maximum generalization capacity without the need to complete the full training cycle, thereby optimizing computational cost and preventing overfitting. **Table 6** summarizes the performance metrics obtained by both models at the point of convergence.

**Table 6:** Performance obtained from the models.

Metric	YoloV8s	YoloV11s
Precision	70.51	73.20
Recall	63.02	66.72
F1-Score	66.55	69.81
mAP@50	70.46	71.63
mAP@50:90	34.49	34.41

The data corroborate that the YOLOv11s architecture offers superior performance in terms of efficiency and operational accuracy. Unlike YOLOv8s, which triggered the Early Stopping criterion at epoch 79, YOLOv11s demonstrated a more sustained learning capacity, extending its training to epoch 97. This additional refinement phase enabled version 11 to better capitalize on complex feature extraction, thus achieving a more sophisticated and robust balance between Precision and Recall.

#### 4.1.1 Performance metrics evaluation

This section describes the dynamic behavior of the models during the training and validation phases. Through the learning curves, the generalization capability of the proposed architectures relative to the baseline is analyzed, allowing the identification of algorithm stability and its convergence toward an optimal solution prior to early stopping (ES).

##### 4.1.1.1 Mean average precision evolution

**Figure 5:**
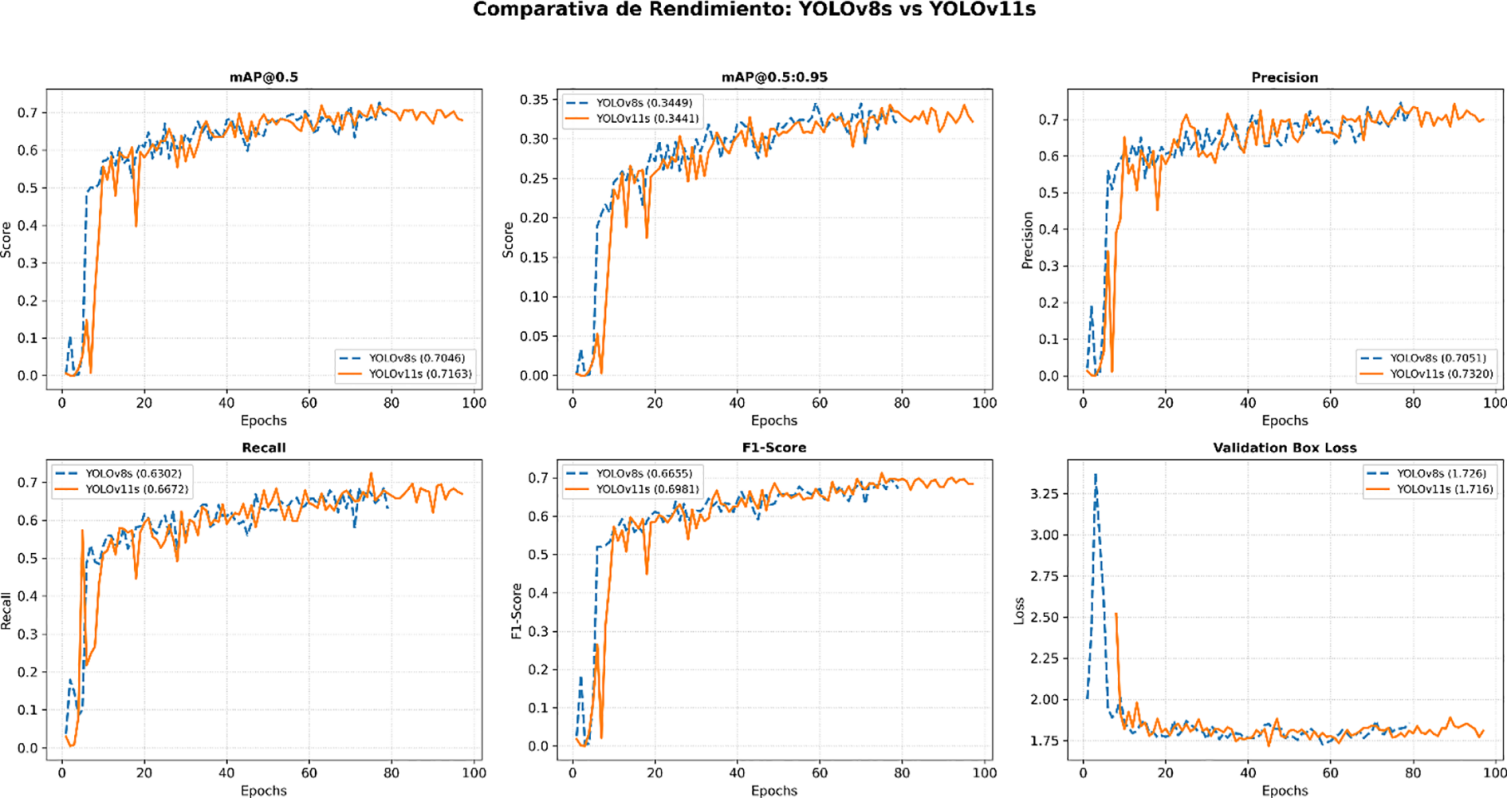
Comparative graph of the obtained results.

The mAP metric was used to quantify the overall accuracy of the object detection system. **Figure 5** presents the evolution of mAP under two Intersection over Union (IoU) thresholds: at 50% and across the 50–95% range.

It can be observed that the YOLOv11s architecture exhibits sustained superiority over YOLOv8s starting from epoch 30. Specifically, under the stricter mAP@50–95 metric, the YOLOv11s curve shows a more stable and upward trajectory, reaching a convergence value of 32.49%, compared to 31.0% for the previous version. This demonstrates that the proposed model not only correctly classifies the presence of the object but also achieves more precise adjustment of bounding box coordinates, reducing spatial error relative to the ground truth labels.

##### 4.1.1.2 Analysis of model behavior: precision vs. recall

To better understand the efficiency of the detectors, a comparative analysis of the Precision and Recall metrics was conducted individually, as illustrated in **Figure 5**. These metrics are fundamental for understanding the trade-off assumed by each architecture between the number of detections and their quality.

Regarding precision, a notable structural difference between both models can be observed. The YOLOv11s architecture demonstrates an ascending and robust learning curve, stabilizing at average values of 0.756, significantly outperforming the 0.735 achieved by the YOLOv8s baseline. This percentage increase indicates that version 11 has a greater capability for extracting discriminative features, enabling it to more effectively filter background **“**noise.**”** YOLOv11s proves to be superior in reducing false positives, avoiding the incorrect classification of empty areas or irrelevant objects as the target class.

Furthermore, the recall analysis at the end of training reveals an overall superiority of the proposed model. Although both models exhibited similar behavior in the early stages, at the early stopping point the YOLOv11s model achieved a Recall of 0.673, surpassing the 0.640 value recorded by the YOLOv8 model.

YOLOv11s did not sacrifice sensitivity to gain precision; on the contrary, it achieved a more robust and stable learning process. While version 8 showed a degradation in its recall capability toward the end of training, the v11s architecture maintained consistency, successfully detecting a greater number of objects.

##### 4.1.1.3 Overall stability (F1-Score)

The behavior of the F1-Score, calculated as previously defined in (Equation 9), is analyzed. This indicator is decisive for validating the technical viability of the system, as it penalizes extreme imbalances between precision and recall. The graphical representation of its temporal evolution throughout training is illustrated in **Figure 5**.

The analysis of the curve evidences the operational superiority of the YOLOv11s architecture, as, when examining the convergence values, it is the proposed model that most effectively mitigates selectivity in recall through outstanding precision, achieving a final F1-Score of 0.712. In contrast, the performance recorded by the v8 model, with its F1-Score limited to 0.68, reveals structural deficiencies in its discriminative capacity at the end of training. This value, situated below the 70% threshold, is mainly attributed to the instability of its precision, which failed to consolidate above 68%. This behavior indicates that the model maintained a high false positive rate, frequently confusing background noise with objects of interest.

##### 4.1.1.4 Validation box loss

The evaluation of the regression loss function allows for the quantification of the geometric accuracy of the predictions. As observed in **Figure 5**, both architectures exhibit a downward trend, indicative of effective learning. However, YOLOv11s demonstrates faster and deeper convergence, achieving a lower minimum loss value than that of YOLOv8s towards the end of training. This metric behavior translates into more precise spatial localization; that is, the bounding boxes generated by the proposed model fit the pest body with greater fidelity, minimizing the spatial margin of error and improving the reliability of automated counting.

### 4.2 Confusion matrix analysis of each model

#### 4.2.1 YOLOv8s

**Figure 6:**
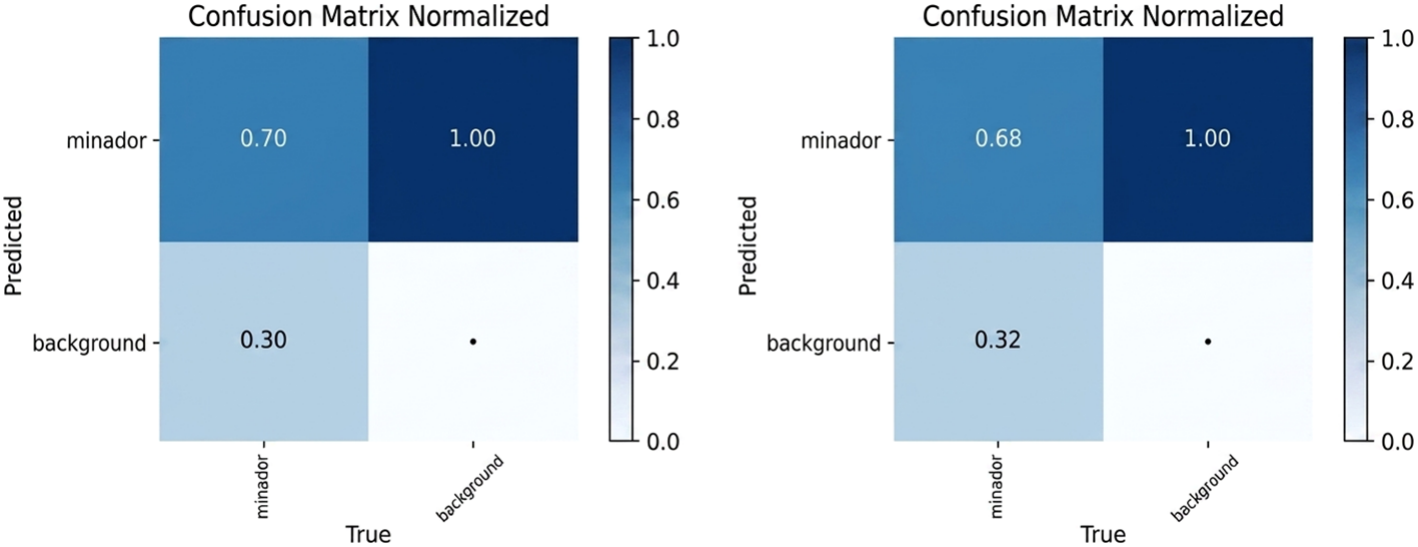
Normalized confusion matrix of YOLOv8s and YOLOv11s.

Regarding the YOLOv8s model, the confusion matrix in **Figure 6** reflects the operational behavior of this architecture under the same testing conditions. This visualization is fundamental for quantifying the impact of its higher sensitivity on the false alarm rate, enabling a direct comparison of detection aggressiveness relative to the more recent v11 version. Specifically, it examines whether the increase in object recall entails a significant cost in the misclassification of environmental elements.

In contrast, the analysis of the YOLOv8s model reveals a critical error pattern in background discrimination. Its recall capacity is reflected in a Recall of 68% (0.68), implying that the model fails to detect 32% of the actual pests (False Negatives). However, the most alarming finding is the massive increase in False Positives: as observed in the upper-right quadrant, the value of 1.00 indicates that the model erroneously classifies all conflicting background samples as ‘leaf miner’. This behavior confirms that the model struggles significantly to distinguish background noise, systematically confusing foliage elements or shadows with the target pest, which severely impacts its overall Precision.

#### 4.2.2 YOLOv11s

The analysis of the confusion matrix for YOLOv11s, as shown in **Figure 6**, breaks down the classifier**’**s performance in terms of true positives and errors associated with false positives and false negatives. Given the nature of small objects in certain leaf miner cases and the high resolution employed, it is crucial to observe how the model discriminates between the complex texture of leaves and the actual pest.

The performance of the YOLOv11s model is characterized by an improvement in detection capability compared to the v8s version. The omission error (False Negatives) has been reduced to 30% (0.30), demonstrating greater effectiveness in identifying the present pest and increasing the Recall to 70% (0.70). However, the challenge persists regarding background discrimination: the matrix again displays a value of 1.00 at the intersection of background and ‘leaf miner’ prediction. This indicates that, although the model better captures actual objects, it maintains a high tendency to generate False Positives, erroneously classifying background textures as the pest—a behavior that must be mitigated in post-processing stages.

### 4.3 Statistical robustness analysis

To overcome the limitations associated with single data split (hold-out) evaluations and ensure the statistical reliability of the results, a 5-Fold Cross-Validation was performed. This method assesses the model’s generalization capability by iteratively training and validating on different dataset subsets, thereby reducing the risk of selection bias.

**Table 7** presents the comparative analysis between the base model (YOLOv8s) and the proposed model (YOLOv11s). The mean (μ) and standard deviation (σ) are reported for each metric, along with the 95% confidence intervals (95% CI) calculated using the Student’s t-distribution. As evidenced in **Table 7**, the proposed YOLOv11s model demonstrates greater statistical consistency. It notably excels in Precision (+1.64%) and mAP@50-95 (+0.70%), while maintaining low standard deviations. The narrow confidence intervals for mAP@50 (for v11s) confirm the model’s stability and robustness against input data variations, validating its suitability for detection under real-world field conditions.

**Table 7:** Statistical performance summary using 5-fold cross-validation (mean ± standard deviation).

Metric	YOLOv8s	YOLOv11s	Difference
Precision	0.712 ± 0.028	0.715 ± 0.029	+0.25
Recall	0.684 ± 0.029	0.663 ± 0.018	-2.05
F1-Score	0.697 ± 0.015	0.688 ± 0.008	-0.95
mAP@50	0.715 ± 0.012	0.707 ± 0.007	-0.74
mAP@50:90	0.344 ± 0.007	0.346 ± 0.007	+0.23

### 4.4 Mobile implementation proposal

#### 4.4.1 System requirements

To ensure the technical and operational feasibility of the proposal in rural environments, the system was designed in compliance with strict functional (FR) and non-functional (NFR) requirements. Regarding its functionality, the application allows for *in situ* image capture and executes inference in a completely offline manner to automatically classify damage caused by the ‘Leaf miner’, visualizing bounding boxes and the confidence percentage on the image. Regarding quality criteria, it was established that the application must be compatible with Android 10.0 or higher and feature a minimalist interface suitable for non-technical users. Furthermore, an operational inference latency suitable for field diagnosis is guaranteed on mid-range devices, displaying only those detections that exceed a 50% confidence threshold.

#### 4.4.2 Application architecture

**Figure 7:**
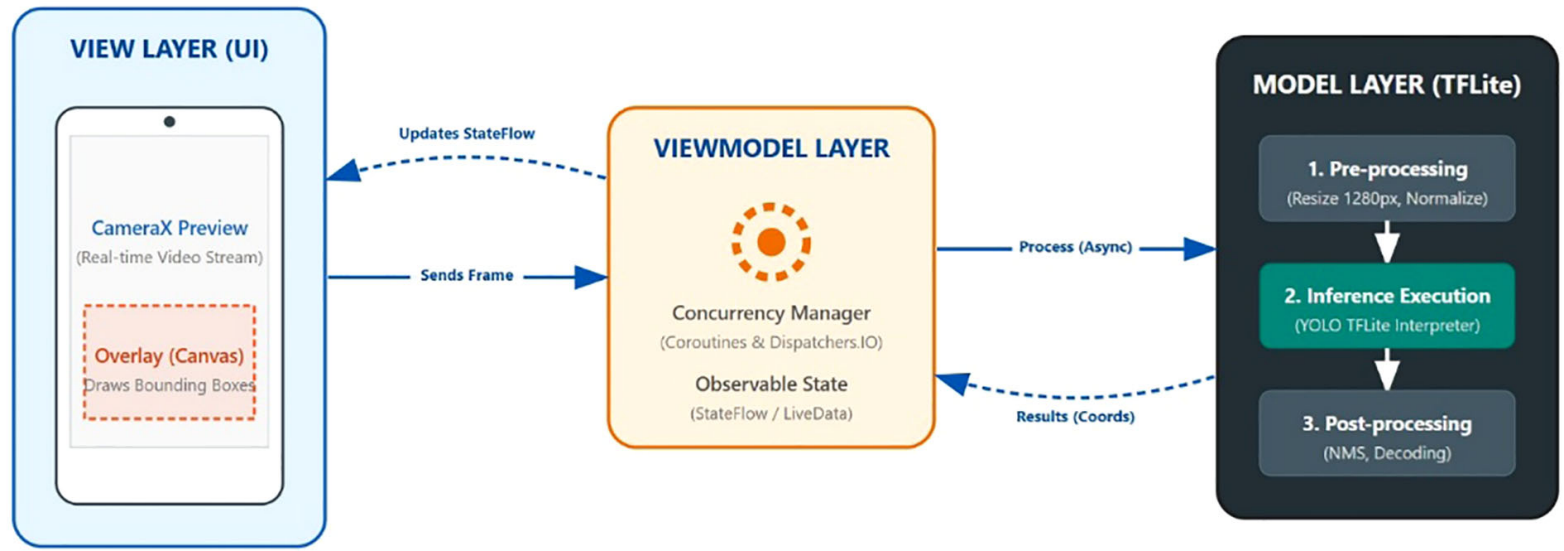
Mobile application architecture.

To ensure optimal performance on mobile devices and maintain code maintainability, a software architecture based on the Model–View–ViewModel (MVVM) design pattern was proposed. This approach enables the decoupling of the user interface (UI) from the image processing logic, the latter being a critical aspect in computer vision applications where model inference must be executed asynchronously to avoid blocking user interaction. A reference illustration is shown in **Figure 7**.

The system was structured into three functional layers:

##### 4.4.2.1 View layer

This layer is responsible for interaction with the farmer, through live capture and/or file-based analysis, representing the two modalities identified for data input into the application. This is achieved through the implementation of the Jetpack Camera library for real-time capture management, or through the image selection module, which allows loading photographs previously stored on the device via the Android API (ActivityResultContracts.GetContent).

Consequently, a graphical view is provided that unifies the visual output. Regardless of whether the image originates from the camera or the gallery, this component renders the bounding boxes and classification labels over the analyzed image.

##### 4.4.2.2 ViewModel layer

This layer operates as the central orchestrator of the system, being responsible for decoupling the user interface from the processing logic and for normalizing the different input sources for the inference engine. It implements a unification logic capable of receiving and processing, interchangeably, real-time video streams (ImageProxy) or static files selected from the gallery (Uri), automatically performing decoding and orientation correction based on EXIF metadata when required. The computational load associated with high-resolution image analysis (greater than 12 MP) is delegated to background threads to prevent user interface freezing.

Consequently, the results of the process are emitted through an observable state implemented with StateFlow, allowing the view to react dynamically and automatically to present graphical detections or notify the user of reading errors.

##### 4.4.2.3 Model layer

This layer constitutes the computational core of the application, where the system analysis resides. YoloDetector is an encapsulated class designed to manage the TensorFlow Lite (TFLite) inference pipeline. Its operational flow begins with a preprocessing stage, responsible for converting images from their native format (camera YUV or Bitmap) to the RGB color space, resizing them to the required input resolution of 1280 × 1280 pixels, and normalizing pixel intensity values. Once the input tensor is prepared, execution proceeds by loading the optimized and quantized model (.tflite) into memory to perform inference. Finally, the process concludes with postprocessing, a stage in which the raw output tensor is interpreted through mathematical algorithms that decode spatial coordinates and apply the Non-Maximum Suppression (NMS) technique, thereby removing redundant detections and filtering out predictions whose confidence probability falls below the established threshold.

#### 4.4.3 Technical specifications

To ensure operational feasibility in Edge AI environments and support the computational load of processing 1280 × 1280 pixel tensors, technical requirements were established based on the validation device (Xiaomi Redmi Note 11). At the hardware level, a processor with 64-bit ARMv8 architecture supporting acceleration (NPU/GPU) and a minimum RAM of 4 GB (6 GB recommended) is required to efficiently manage heap memory during execution. Likewise, it is necessary to have available storage space for the APK and quantized models, along with a rear camera of at least 12 MP (50 MP recommended) compatible with the CameraX API for high-quality frame extraction. Regarding software, deployment requires an Android 10.0 or higher operating system and is based on native development (Kotlin) to maximize hardware integration. Finally, inference execution utilizes the optimized TensorFlow Lite interpreter, capable of processing compressed YOLOv8s or YOLOv11s models with low latency.

#### 4.4.4 Inference workflow

Through a standardized sequential process that transforms a conventional image into useful agronomic information, the steps of the workflow are as follows:

##### 4.4.4.1 Image acquisition

The user captures a photograph of the crop using the application’s camera interface. The image is obtained in its original high resolution.

##### 4.4.4.2 Preprocessing

In this stage, the input whether the full image or a region of interest, is resized to the spatial resolution required by the model’s input tensor (1280 ×1280). Subsequently, linear normalization is applied, transforming pixel intensity values from the range [0, 255] to the floating-point range [0, 1].

##### 4.4.4.3 Model execution

At this point, the processed tensor is input into the (TFLite) interpreter. The model performs forward propagation to predict coordinates, classes, and confidence scores.

##### 4.4.4.4 Postprocessing

The raw model outputs are converted into bounding box coordinates. In conjunction with Non-Maximum Suppression (NMS), redundant detections are filtered, whereby boxes with confidence scores below the established threshold are discarded and excessive overlaps are eliminated.

##### 4.4.4.5 Visualization

Valid detections are drawn on the scaled original image, displaying the colored bounding box (Leaf miner).

### 4.5 Mobile device performance evaluation

To validate applicability in low-resource scenarios, the quantized models (TFLite format) were deployed on a mid-range device (Redmi Note 11, Snapdragon 680). Average performance was measured over independent runs processing native inputs of 1280 × 1280 pixels. As observed in **Table 8**, the proposed YOLOv11s model demonstrated superior performance, recording an average inference time of 5.28 s with a marginal standard deviation of ± 0.13 s. This low statistical variability confirms the model’s stability compared to YOLOv8s (6.19 ± 0.40 s), achieving a latency reduction of ~15%. Memory consumption remained stable at ~116 MB, validating that the proposed architecture is highly efficient for high-resolution *in situ* diagnostics without compromising hardware resources.

**Table 8:** Performance comparison on mobile device.

Evaluation paramenter	YOLOv8s	YOLOv11s
Input Size (px)	1280×1280	1280×1280
mAP@50 (%)	71.2	71.5
Inference Time (s)	6.19 ± 0.40	5.28 ± 0.13
Memory Usage (mb)	113.5	116.3

## 5 Discussion

This research addressed the detection of the leaf miner in sweet potato crops using deep learning algorithms, demonstrating that the evolution of YOLO architectures allows for the identification of complex foliar damage with limited computational resources. The obtained results evidence that the YOLOv11s model outperforms its predecessor, YOLOv8s, achieving a Precision of 73.2% and a mAP@50 of 71.63%, compared to 70.51% and 70.46% for the base model, respectively.

Upon contrasting these results with existing literature, notable differences in precision metrics are observed, which depend on the nature of the pest and the environment. For instance, research such as that conducted in (Kini et al., 2024) reported a precision of 99.67% using CNN (ResNet-18) for pathologies in pepper leaves, and in (Chand et al., 2023a), 97.5% was achieved with ResNet for whitefly detection. Although these values are higher than those obtained in our study (73.20%), it is fundamental to consider that leaf miner detection presents a greater visual challenge: unlike whole insects or circular disease spots, minor damage consists of irregular serpentine galleries that camouflage with the leaf venation and the complex background of the open-field crop. Furthermore, models such as ResNet typically require higher computational cost, whereas our proposal prioritized “Small” architectures (v8s and v11s) viable for mobile devices.

Regarding the use of the YOLO family, previous studies such as (Ahmad et al., 2022) achieved an mAP@0.5 of 98.3% using YOLOv5x to identify pests via smartphones. However, said study employed the “Extra Large” (x) version of the model, which demands significant hardware resources that are not always available to the average smallholder farmer. In contrast, our research demonstrates that by using lightweight “s” versions, the YOLOv11s model achieves an efficient balance, maintaining a competent generalization capability (F1-Score of 69.81%) without sacrificing implementation viability on mid-range devices, a critical need for agricultural technification in rural areas.

A relevant finding of this study was the capability of YOLOv11s to reduce False Positives compared to YOLOv8s. While version 8 exhibited more aggressive behavior with greater confusion with the background, as observed in the confusion matrices; the v11s architecture, thanks to the incorporation of C3k2 blocks and the C2PSA attention module, achieved more robust discrimination between healthy tissue and miner galleries. This contrasts with older or more generalist approaches which, as mentioned in (Yonbawi et al., 2023), require intensive image preprocessing to achieve reliable classifications. Our methodology validates that the intrinsic architectural improvements of version 11 allow for omitting heavy preprocessing, facilitating real time inference.

Finally, the integration of data augmentation techniques such as mosaic, mixup, and rotation proved indispensable for mitigating overfitting, given that the work was conducted with a limited initial dataset. This aligns with what is presented in (Jabir and Falih, 2021), where regularization techniques were key to performance in weed and crop environments. Unlike methods that depend exclusively on laboratory conditions, our model has been trained and validated with field images that include variations in lighting and background, suggesting that, despite having lower numerical metrics than studies in controlled environments, the proposed system possesses real practical applicability for supporting sweet potato crop monitoring.

## 6 Conclusions

Regarding the dataset, the “camote_minador” dataset was successfully designed and structured, initially composed of 350 high-resolution images captured in the districts of Chosica del Norte and Callanca. Through the application of data augmentation techniques (rotation, mosaic, mixup, and HSV variations), the diversity of the training corpus was increased, which was decisive for the models to learn to recognize complex patterns such as serpentine galleries and necrosis under varying field conditions, thereby avoiding overfitting in a moderate sized dataset.

Regarding the training, the comparative analysis evidenced that the YOLOv11s architecture offers superior performance for pest detection compared to its predecessor, YOLOv8s. Thanks to the integration of C3k2 blocks in the backbone and the C2PSA attention module, the v11 model achieved a more stable convergence and improved feature extraction for small objects, validating the hypothesis that recent architectural improvements directly influence the ability to discriminate between foliar damage and background noise (healthy foliage or soil).

In the evaluation of metrics, the quantitative assessment determined that the YOLOv11s model achieved a Precision of 73.20% and a Recall of 66.72%, surpassing the metrics of YOLOv8s, which obtained 70.51% and 63.02%, respectively. Likewise, the proposed model achieved an F1-Score of 69.81%, demonstrating a more robust balance between pest detection capability and the minimization of false alarms, establishing itself as the most effective alternative for monitoring the leaf miner in sweet potato crops.

The technical feasibility for the implementation of a mobile prototype based on the MVVM architecture and the TensorFlow Lite interpreter was established. Requirements analysis and efficiency tests confirm that it is possible to execute the optimized model on mid-range devices (with 4GB of RAM or more) without reliance on internet connectivity, meeting the response times necessary for the farmer to conduct *in situ* inspections in an agile and autonomous manner.

## Data Availability

The raw data supporting the conclusions of this article will be made available by the authors, without undue reservation.
